# Early testing of new sanitation technology for urban slums: The case of the Blue Diversion Toilet

**DOI:** 10.1016/j.scitotenv.2016.10.057

**Published:** 2017-01-15

**Authors:** Robert Tobias, Mark O'Keefe, Rahel Künzle, Heiko Gebauer, Harald Gründl, Eberhard Morgenroth, Wouter Pronk, Tove A. Larsen

**Affiliations:** a Eawag, P.O. Box 611, 8600 Dübendorf, Switzerland; b EOOS Design, Zelinkagasse 2/6, 1010 Vienna, Austria; c ETH Zürich, Stefano-Franscini-Platz 5, 8093 Zürich, Switzerland

**Keywords:** BDT, Blue Diversion Toilet, BMGF, Bill & Melinda Gates Foundation, MBR, membrane bioReactor, RTTC, Reinvent The Toilet Challenge, SI, supporting information, UDDT, Urine Diverting Dry Toilet, UGX, Uganda Shilling (currency used in Uganda), Urine diversion, Gravity-driven membrane bioreactor, Innovation, Usability, Acceptance, Low-income countries

## Abstract

The toilets used most in informal urban settlements have detrimental consequences for the environment and human health due to the lack of proper collection and treatment of toilet waste. Concepts for safe, sustainable and affordable sanitation systems exist, but their feasibility and acceptance have to be investigated at an early stage of development, which is difficult due to the high costs of building working models. In this paper, we present an approach to estimate acceptance in a valid and representative form with only one working model, and apply it to test an innovative zero-emission toilet with recycling of wash water. Four basic principles were specified for investigation and nine hypotheses formulated to test the feasibility and acceptance of these principles: source separation of urine and feces with subsequent collection for resource recovery; provision of wash water in a separate cycle with on-site recovery through a membrane bioreactor; a convenient and attractive overall design; and a financially sustainable business plan. In Kampala (Uganda), in 2013, data was collected from 22 regular users, 308 one-time users and a representative sample of 1538 participants. Qualitative data was collected from the users, who evaluated their likes, perceived benefits, social norms and expected ease of use based on verbal and visual information. Most of the hypotheses were confirmed, indicating the feasibility and acceptance of the basic principles. Source separation and on-site water recovery were found to be feasible and accepted, provided users can be convinced that the emptying service and water recovery process work reliably. In the survey, the toilet was evaluated favorably and 51% of the participants agreed to be placed on a bogus waiting list. However, some design challenges were revealed, such as the size of the toilet, hiding feces from view and improving the separation of urine and water.

## Highlights

• Inadequate sanitation in urban slums is a threat to the total environment.
• Zero-emission toilet with recycling of wash water tested in Slum in Kampala, Uganda.
• Approach for valid & representative acceptance estimate with only one working model.
• Source separation & on-site water recycling is feasible and has market potential.
• Separating urine from water and the feces compartment pose challenges to the design.

## Graphical abstract

**Figure:**
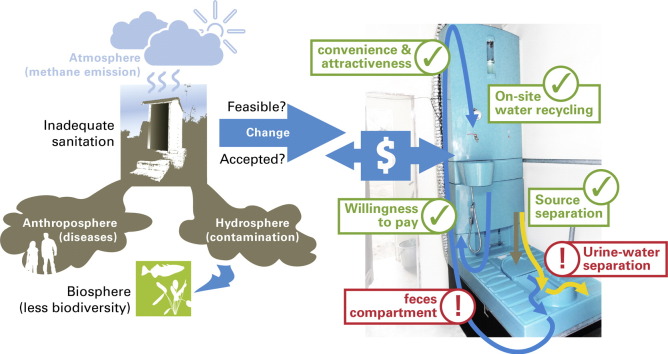

## 1 Introduction

Worldwide, 2.4 billion people live without adequate sanitation (United Nations, 2015), a situation which has grave implications for public health and the environment. Diarrhea – mostly caused by unsafe sanitation and drinking water, combined with a lack of hand-washing with soap – kills about 760,000 children under five annually (World Health Organization, 2013). Particularly in the informal urban areas of low-income countries, it has proved extremely difficult to develop adequate sanitation systems that safely contain, transport, treat and dispose or reuse waste.

The typical toilet technology in urban slums consists of pit latrines (Jenkins et al., 2015): this has a number of negative consequences for public health as well as the environment. Emptying is often unhygienic and expensive, leading to dangerous practices of overfilling the pit and/or flooding it out (Jenkins et al., 2015). Ground water contamination is frequent (Graham and Polizzotto, 2013), and in many cases the fecal sludge is dumped into water courses, with devastating effects on surface water quality (Semiyaga et al., 2015). Increasing eutrophication in low and middle income countries, to a large degree caused by the nutrients contained in human excreta from cities (Nyenje et al., 2010), is raising awareness that the sanitation crisis is detrimental not only to public health, but also to the environment. This is reflected in the more comprehensive Post Millennium Development Goals for sanitation, which also involve water pollution control and resource recovery (UN Water, 2015), and are discussed in more detail in Larsen et al. (2016).

Whereas the effects of pit latrines on water quality are well documented (see above), only little information is available on the possible effects on climate. However, with close to two billion people relying on this technology (Graham and Polizzotto, 2013), methane emissions could be substantial. Yearly methane emissions are estimated to be around 1 kg per person from the anaerobic processes in pit latrines (Reid et al., 2014), corresponding to about 2% of the methane emissions from an average person.

In urban slums, it is not an easy task to find alternatives to pit latrines, and more attractive on-site technologies are often considered merely a temporary solution until off-site sanitation can be afforded in the slum (Kerstens et al., 2016). The low-cost sewers suggested in (Paterson et al., 2007) as the most appropriate sanitation technology for low income, high-density urban areas fail to offer adequate solutions for water provision and water pollution control. Katukiza et al. (2010) identified the Urine Diverting Dry Toilet (UDDT) and biogas latrines as possible good solutions for a specific urban slum in Uganda, but point out the lack of acceptance of these simple technologies. Furthermore, neither solution represents an integrated option for hand-washing, a necessary element for making any sanitation solution truly hygienic (Greenland et al., 2013).

In 2011, the Bill & Melinda Gates Foundation (BMGF) challenged a number of research institutions to find more complete sanitation solutions for the urban poor living on less than US$2 a day (Reinvent The Toilet Challenge, [RTTC]) (Anonymous, 2011). The BMGF called for high user comfort, zero emissions to the environment, on-site solutions for resource recovery, and low costs of US$0.05 per person per day. The costs are comparable to the lifecycle costs of community-based sanitation solutions with simple anaerobic technologies reported for Indonesia by Kerstens et al. (2015) (US$0.03/p/day), but should provide significantly more comfort for the users. This goal should be achievable in a typical slum area with no grid infrastructure (no electricity, piped water or sewers).

The Blue Diversion Toilet (BDT) was developed as part of this program (Larsen et al., 2015). The BDT is essentially a UDDT as suggested by Katukiza et al. (2010), improved with a separate water cycle (the ‘blue’ diversion) for personal hygiene (hand-washing, anal cleansing, and menstrual hygiene) and flushing of the front compartment. It combines the simplicity of the water pollution control and resource recovery of a UDDT with the hygienic advantages of an integrated hand-washing facility. Zero emission and grid independence are ensured via internal water treatment and recycling (Künzle et al., 2015).

The BDT is based on the Sustainability Development Goals (SDG), and helps fulfill the requirements described primarily in SDG 6: providing safe sanitation with hand washing with a special emphasis on menstrual hygiene (SDG 6.2), saving water through a closed water cycle (SDG 6.4), and preventing water pollution through the zero-emission principle (SDG 6.3). As far as possible, the BDT concept follows the principle of SDG 11 (sustainable cities): Most parts of the toilet can be produced locally (the plastic parts, for instance, by the simple process of rotational molding), and the service and resource recovery concept provides local job opportunities.

Resource recovery is the basic concept of ecological sanitation leading to highly efficient water pollution control because the pollutants are turned into valuable products instead of being discharged to the environment. On average, the yearly excretion of a human being amounts to 22 kg COD (organic matter) with an energy content of 270 MJ, 3.7 kg nitrogen (N) and 0.7 kg phosphorus (P). The BDT concept allows > 95% of these resources to be recovered.

These ambitious sustainability goals can only be fulfilled by a specific design that differs considerably from that of the typical aspirational flush toilet. This calls for co-design with potential users. Furthermore, including a water cycle imposes costs on users and developers alike, and these will only be justifiable if the availability of clean water helps transform the rather unattractive UDDT into an aspirational product. While established market research techniques are well suited for testing the development of incremental innovations to an already existing product (Leifer et al., 2000, Lynn et al., 1996), it is a greater challenge to assess more fundamental innovations (Lettl, 2007, O'Connor and McDermott, 2004). Most importantly, it is difficult for potential users to imagine a product that does not yet exist – something which is required in order to provide valid evaluations (Veryzer, 1998).

A framework for including users in the development of such innovations in the medical-technology sector (Lettl, 2007) includes three aspects: (1) identification of the development stage and purpose for engaging with users; (2) close interaction with competent “lead users” who are likely to purchase the product; and (3) ensuring that many users interact with the product. Our study examines the development phase (i.e. after ideation, but before market testing), and the purpose of engaging with users is to test the feasibility and acceptance of the basic design principles. To interact with lead users, it is necessary to test the toilet in the target area (i.e. urban slums). The third aspect is more problematic in this case. For practical reasons, only one working model of the BDT could be produced, which limited the number of people who could test it.

We propose an approach for testing fundamental innovations at an early stage of development (i.e. long before the final product is specified) when it is not possible or efficient to build a large number of working models. The first step of this approach is to define the design principles at a rather abstract level (since the details of implementing these principles are not yet known). For each design principle, tests are created that are specific enough to obtain conclusive results and identify causes of any failures and can thus serve as a basis of further improvements of the technology. A mix of social-scientific methods is then used to obtain a valid, reliable and representative picture of how applicable and accepted the principles are.

Here, we apply this approach to test the BDT at an early stage of its development. The aim of this exercise is twofold. On the one hand, we investigate a number of basic principles relevant for innovations in the sanitation sector in developing countries. On the other hand, we want to illustrate the approach so that it can be applied to other fundamental innovations developed within relevant research projects with a limited budget.

## 2 Design principles of the Blue Diversion Toilet and the testing procedure

In a first step, abstract design principles are formulated and specific tests developed in order to evaluate them. Each test consists of a hypothesis and criteria that specify the conditions under which the hypothesis is supported or not by the data. Here, we present the principles and tests specific to the BDT. All hypotheses tested and the criteria used for testing are summarized in Table 1.

*Principle 1: source separation and resource recovery.* As discussed in Section 1, a resource-recovering UDDT was chosen as a model for the BDT. Since a number of processes exist for medium-scale resource recovery from source-separated toilet waste (McConville et al., 2014), we propose a system for the safe manual collection and transport of source-separated feces and urine to a local resource-recovery plant serving about 1000 people (Schmitt et al., 2016).

*Principle 2: provision and recovery of water for washing.* An important problem of conventional sanitation in urban slums is the lack of hygiene facilities. Hand-washing with soap is the most effective measure against diarrheal disease (Mattioli et al., 2013), and is also effective against respiratory illnesses (Curtis et al., 2011). Further, options have to be provided for anal cleansing (required for Hindus and Muslims) and menstrual hygiene – both serious challenges in urban slums. Since clean water is not available in many urban slums and the goal is a zero-emission toilet, water has to be separated from feces and urine, then recovered and reused within the toilet (Künzle et al., 2015).

*Principle 3: convenient and aspirational design.* The overall design of the toilet not only has to be convenient but also has to suit the cultural norms and habits of the target population. Considering the relatively high price for its intended users, the design has to make the toilet an aspirational (i.e. desirable and social-status enhancing) asset so that consumers are more likely to purchase it (Larsen et al., 2015).

*Principle 4: sustainable business model.* Various business models are available for the provision of water-related services in low-income countries. In this case, a franchising model of service provision by local entrepreneurs based on a weekly or monthly fee was chosen (Gebauer and Saul, 2014).

**Fig. 1:**
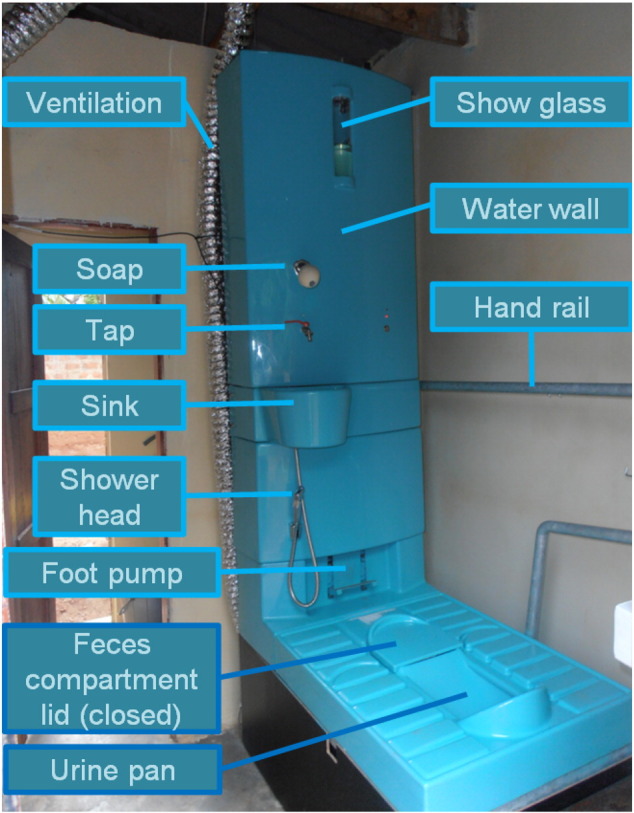
The working model of the Blue Diversion Toilet as used in the Kampala field test. The water wall contains the water tanks and bio-reactor. The metallic tube (labeled ventilation) is part of the active ventilation of the feces compartment to prevent odor.

The four principles and their testing within the working model (Fig. 1; see also supporting information, SI, Section 1.1) are described in more detail in the following sub-sections.

### 2.1 Principle 1: source separation and resource recovery

Urine contains most of the nutrients in wastewater, while energy and organic matter can be recovered from feces. A large number of technologies exist for resource recovery from urine and feces (Larsen et al., 2013). Source separation in the form of UDDTs is frequently used in low-income countries because it reduces odor, but user acceptance is low (Cordova and Knuth, 2005, Katukiza et al., 2010). This is partially due to lower user comfort and the unhygienic appearance of the UDDTs, but the collection of urine and feces is also often left to the users, whereas pits are emptied by professionals (Jackson, 2005). A pan was consequently designed for comfortable use, and water is provided for flushing the pan, anal cleansing, and menstrual hygiene. To prevent urine and feces being diluted by water when the flush or shower head (for anal cleansing and menstrual hygiene, see Fig. 1) is activated, a lid automatically closes the feces compartment and the valves below the pan are switched to the position for collecting the water for recovery. Finally, a service that empties the toilet twice a week is part of the BDT business plan (see Principle 4 section). The use of relatively small containers allows the toilet to be installed on elevated platforms, making the design suitable for flood-prone areas. For this principle, two hypotheses are tested:

H1a Source separation (as a prerequisite for resource recovery) is technically feasible for toilets in urban slums. Two criteria are used for testing this hypothesis: (1) the amount of solid excreta in urine and water and (2) the amount of urine in the water. Criterion (1) indicates that (a) the separation of urine and feces complies with the toilet practices of the users, and (b) that the pan and platform are well-designed. Some contamination is, however, possible (and tolerable) due to anal cleansing with water. Criterion (2) indicates that the separation of water and urine works correctly.

H1b Inhabitants of the investigated slums accept source separation and resource recovery. The criteria for testing this hypothesis are the percentage of negative user comments on the principle of separating feces from urine, the design of the pan and feces compartment, and the fact that the toilet requires an emptying service. A small percentage of participants (specified here as < 20%) giving negative comments indicates acceptance of these principles.

### 2.2 Principle 2: provision and recovery of water for washing

The BDT provides water for flushing, personal hygiene, and hand-washing. All used water is recovered and reused within the toilet. Even though wash water does not have to meet drinking-water standards, it must be microbiologically safe to avoid infections (e.g. due to open wounds). Furthermore, for recycled water to be accepted, we hypothesize that it should be colorless and odorless.

We developed an aerated, gravity-driven membrane bioreactor (MBR) for on-site water recovery without the need for regular maintenance (Künzle et al., 2015). The low-loaded ultrafiltration membrane is kept permeable by biological activity in the biofouling layer (Derlon et al., 2012, Peter-Varbanets et al., 2010) and is integrated into the water wall at the back of the BDT (see Fig. 1). The design was tested at temperatures between 15 and 25 °C (Künzle et al., 2015). In hot countries, shading and perhaps some evaporative cooling are required to keep the water temperature acceptable for humans as well as for the biological activity in the reactor. Membrane filtration produces pathogen-free water, but a subsequent polishing step is required to remove color and prevent bacterial regrowth (see SI, Section 1.1).

The hypotheses tested for this principle are as follows:

H2a On-site water recovery for washing is technically feasible for toilets in urban slums. The principal criterion (Criterion 1) for testing this hypothesis is the microbial quality of the wash water, which has to be unproblematic at all times. Additionally, the color and smell of the treated water are used as a criterion, and should be minimal (Criterion 2).

H2b The inhabitants of the investigated slums accept the fact that recycled water is used for hand-washing and personal hygiene. The percentage of participants giving negative comments on using recycled water for washing (< 20%) is used as a criterion for testing this hypothesis.

H2c The provision of water for washing is an important selling point for new sanitation technologies in urban slums, justifying expensive additional components for water treatment. This hypothesis is tested with the percentage of positive comments on the water-related features. Since positive aspects are, in general, less frequently noted and, thus, commented, the criterion is set to 10% of the participants giving positive comments on these features.

### 2.3 Principle 3: convenient and aspirational design

Designers from the Austrian design office EOOS developed the toilet interface on the basis of the results of self-tests and group discussions with slum inhabitants in Kampala. To achieve a convenient and attractive toilet, an L-shaped structure was chosen. The structure consists of a platform with a squatting pan and a vertical part where the water tanks are stacked and popular bathroom features (hand-washing facility, a shower head for personal hygiene, and a flushing mechanism) are provided (Fig. 1). Users pump clean and polluted water in parallel into the clean water tank and treatment reactor respectively. For odor control, we used active ventilation (i.e. a small ventilator that produces a vacuum in the feces compartment; see Fig. 1). For the three months of field testing, no technical fly filters were installed.

To measure the convenience and attractiveness of the BDT, two hypotheses were tested:

H3a The BDT is more attractive than any other sanitation option available to the persons testing the BDT. This hypothesis is tested on the basis of indicators as to how much the toilet was actually used, derived from measurements of water usage and collected feces and urine. For ten persons, it is expected that a maximum of about 10 L of urine and 2.1 kg of feces will be produced per day and up to 75 L (1.5 L per toilet visit and 50 toilet visits per day) of water will be used. In particular, the amount of feces should not be considerably lower (Criterion 1), since the most important use of a toilet such as the BDT is in the morning for defecating. Further, the use of water should be proportional to the use of the toilet (Criterion 2), indicating that the wash water is actually used when the toilet is visited.

H3b The design of the toilet is convenient for the inhabitants of the investigated slums. The criterion for testing this hypothesis is the percentage of negative comments on the usability of the toilet (should be < 20%).

### 2.4 Principle 4: sustainable business model

The business model (Shafer et al., 2005) was developed in parallel with the toilet design. Since the actual operation and maintenance exceed the core competencies of toilet manufacturers, whose rationale is simply to sell as many toilets as possible, a sanitation service provider specializing in operating and maintaining the toilets was considered as the most promising business model. The toilet is not sold but rented to the users, which is an unknown concept in the investigated areas. Since most users rent houses, it is assumed that the toilet would usually be rented by landlords, but the rental fee would be added to the rent for the houses. Renting has the advantage that people do not face any upfront investments. The rental fee of 0.05 US$/p/day (15 US$/month for 2 families of 5 each) covers the capital cost (production and installation), toilet emptying and maintenance, and the transport and treatment of excreta, but also allows a profit of US$ 0.015/user/day (the assumptions are compiled in SI, Section 1.4). For about the price of one use per person and day of a public toilet (US$0.07/use according to our own experience in the region), the users get a toilet at home that provides wash water, has an aspirational and functional design and does not smell. To estimate the population's willingness to pay this price, but without having a product to sell as yet, we tested two hypotheses:

H4a The psychological determinants of purchase decisions are favorable. The criteria for testing this hypothesis are (1) to have at least one third of the responses in the highest two answering categories and (2) less than one third of the answers should be negative or neutral or, in the case of unipolar items, in the lowest two answering categories.

H4b Simulated purchase decisions are positive for a sufficiently large percentage of people. As a criterion we defined that at least one third of the participants should decide in favor of renting a BDT.

The psychological determinants are derived from the theory. A common approach (see e.g. Tobias and Berg (2011) for a similar investigation of an innovation in the drinking-water sector) is to build on the theory of planned behavior (Ajzen, 1991), but to further specify its components. This theory explains decisions on the basis of attitudes (how good or bad an option is), subjective social norms (consequences for one's reputation), and perceived behavioral control (ease of implementing or living with an option). Attitudes are further categorized into affective (liking an option) and instrumental (cost-benefit analyses—here, health effects) (Breckler and Wiggins, 1989). These factors also reflect some critical characteristics of innovations mentioned by Rogers (1995), in particular the relative advantage (instrumental attitudes) and complexity (perceived behavioral control). Further, the compatibility of the toilet will be investigated on the basis of comments about it. The other factors mentioned by Rogers (1995) – testability, potential for reinvention, and observed effects – cannot be investigated at this early stage of development. The overall evaluation (how much better or worse it would be to have/use a BDT) and the intention to rent a BDT are also assessed.

The valuation alone is not indicative of the intent to rent a toilet. Constraints such as financial uncertainties, lack of space, and not being in charge of such a decision might hinder a person with a high valuation from renting the toilet (O'Keefe et al., 2015). In contrast, a lack of alternatives may even make a person with a low valuation of a product decide in its favor. We therefore simulated a purchase decision using an item that offers the participant the choice of being placed on a waiting list, i.e. as soon as the toilet became available, they would have to pay UGX 30,000 (US$11) per month for it and would have the toilet and services delivered. After giving their answer, the participants were told that the waiting list was not real. The price used in this item takes into consideration the smaller household size in the target area (for the derivation of this price and further information on this item, see SI Section 2.5). All hypotheses and the criteria tested are summarized in Table 1.

## 3 Methods

The field test was performed from April to July 2013 in two slums (Kifumbira and Kisalosalo) in Kampala, Uganda. For the technical and psychological pretests, the toilet was first installed at Makerere University and used by its academic and non-academic employees. The toilet was then set up in a community center in Kifumbira and was used for three weeks during workshops. It was finally installed in a superstructure in Kisalosalo for regular use by selected households. Technical data relating to water consumption and water quality were recorded at different time intervals as described in SI, Section 1.3.

To investigate the first three principles, we used a qualitative approach (Berg and Lune, 2013), mainly because we did not know which aspects might be critical and to avoid biases due to forced evaluations (see SI, Section 2.3). This approach also allows us to assess evaluations based on more unconscious processes, as the participants can also express themselves rather vaguely (e.g. something does not feel right). To investigate Principle 4, quantitative data were gathered because the evaluation dimensions were pre-defined (on the basis of psychological theory), and a quantitative assessment based on more conscious evaluations was therefore preferable (see SI, Section 2.4).

We used three different samples to maximize the validity and representativeness of the data with only one working model available. The most valuable data came from persons who used the toilet regularly over a longer period of time. With the single working model available, we gathered data from 22 members of six households each of whom used the toilet for two weeks (two households at a time). The participating households were selected on the basis of the following criteria: closeness to the toilet, willingness to participate, composition of the household, cultural toilet habits (washer/wiper), and religious background. The regular users were interviewed at home five times during the two weeks they used the toilet. The first three principles were investigated on the basis of comments.

To obtain a more representative and complete set of evaluations, a stratified sample (men/women; wiper/washer; women with children; elderly and disabled persons) of 308 persons was invited to use the toilet once at the community center in Kifumbira. After using the toilet, semi-structured private (i.e. only one participant and one interviewer were present) interviews focusing on the toilet's usability were performed. These qualitative data were used to investigate the first three principles.

Finally, a representative survey was conducted with 1538 persons, sampled with the random-route method (i.e. the interviewers walked along a predefined route through the target area and asked a randomly selected person from every third household whether he or she would be willing to participate). Of the persons contacted, 27% declined to participate, mostly due to a lack of time. This sample was considered as representative of the population (Hoffmeyer-Zlotnik, 2003). The participants evaluated the toilet and related services on the basis of information provided by the interviewers, including photos and diagrams (see SI Section 2.2). Open comments were used to investigate the first three principles. Closed questions used for assessing psychological determinants and the bogus waiting-list item were used to investigate Principle 4.

The wording of all items and further information on the questionnaires are provided in the SI (Section 2.3 to 2.5). The data were analyzed using descriptive statistics. Open answers were categorized using the direct content analysis method (Hsieh and Shannon, 2005): the number of times each category was mentioned was calculated as a percentage. More details on the categorization of the open items are given in the SI (Section 2.6), including sample quotes. For the closed questions, frequencies of answers are presented.

The study was conducted in strict compliance with the ethical principles of the American Psychological Association and the Declaration of Helsinki. Ethical approval was granted by the ethics review board of the University of Zurich, Switzerland, on April 16, 2013. Written, informed consent was obtained from all participants, and in the case of children, also from their parents. Further information on the measures taken to ensure the participants' comfort is available in the SI (Section 2.1).

## 4 Results

**Fig. 2:**
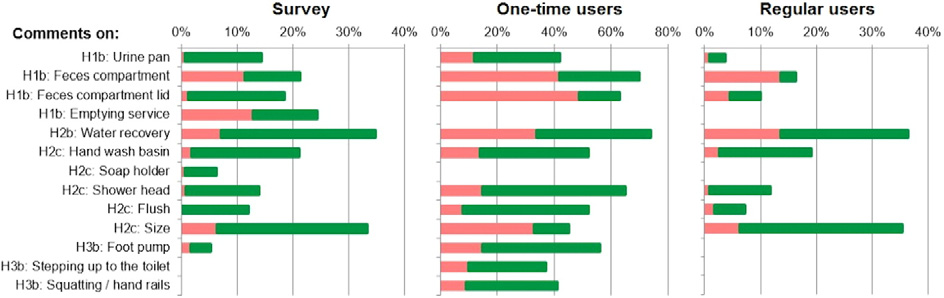
Percentages of persons of each sample mentioning features in negative (light red) and positive (dark green) comments in reply to open questions. Note: The absence of a bar means that no comments were given on a feature by this sample.

The toilet was in operation for 70 days and was used about 1500 times. With only little maintenance (see SI Section 1.2), all parts of the user interface functioned without problems during the entire test. Also, no problems with odor or flies were reported. Fig. 2 presents the percentages of positive and negative comments obtained by using open questions (see SI, Section 2.3) on various features from the three samples (survey, one-time users, and regular users). Numerical values (Table S4) and sample quotes (Table S3) are provided in the SI.

### 4.1 Principle 1: source separation and resource recovery

An almost total absence of feces in water and urine indicates that the separation of liquid and solid excreta is feasible in the study area (H1a, Criterion 1). However, the urine-to-water ratio in the water-recovery system (H1a, Criterion 2) was more than ten times above the expected amount, resulting in the treated water having a bad odor. As a temporary solution, the odor was removed by adding an activated carbon filter. The failure of the urine-water separation process (H1a, Criterion 2) is at least partially due to the large amount of mud (including dust that turned to mud in the toilet's own water) that was flushed into the separation mechanism. This is an additional challenge for separating urine and water under slum conditions.

Regarding Hypothesis H1b, the separation of urine and feces was not mentioned frequently, even though the urine pan itself tended to receive positive comments (Fig. 2). In contrast, the feces compartment was one of the most criticized components and 42% of the one-time users gave negative comments on it. In particular, the compartments were perceived as being too small —people feared that they would fill up too quickly, and they did not like to be so close to the feces or to see them. The emptying service was another feature in the survey with a high proportion of negative comments, though the 13% of survey participants giving such comments are still under the 20% specified as a criterion (H1b). Due to their experiences with other services, or rather the lack of functioning services, the users did not believe that a reliable collection service was possible. Finally, the one-time users also did not like the feces compartment lid, which fell off easily (49% of them gave negative comments on it). The lid had been designed to be easily removable for cleaning, but it often fell off when being opened. In contrast, the survey participants positively evaluated the existence of a feces compartment lid. Overall, the principle of source separation appears to be accepted by the population. However, the design needs improvement, and promoting the emptying service will present a major challenge.

### 4.2 Principle 2: provision and recovery of water for washing

From a technical perspective, apart from the separation-mechanism problems mentioned in Section 4.1, the water-recovery system worked well, and the microbial quality of the treated water was unproblematic during the entire test period (H2a, Criterion 1). Furthermore, > 10% of the participants gave positive comments to all water-related features (except for the soap holder and, for the regular users, the flush) (H2c, Fig. 2). Thus, the water-related features appear to be a crucial selling point. The idea of having safe water without refilling was welcomed, but there were doubts about the water quality. Most of the survey participants evaluated the idea of recovered water positively, while one-time and regular users had mixed feelings about it, primarily due to the odor problems. For the one-time users, the criterion for H2b was not fulfilled: 34% gave negative comments. However, 40% of this group gave positive comments on this principle. Thus, provided that the problems of urine–water separation can be solved, water recovery on-site appears to be feasible and accepted by the population. However, when promoting the toilet, a critical point will be to convince people that they can trust the safety of the recovered water.

Another consequence of storing and recovering water within the toilet is its size. The one-time users were especially disturbed by this aspect (33% gave negative comments, Fig. 2), because they experienced the size directly and compared it to the superstructures used for their latrines. In contrast, the regular users and the survey participants tended to evaluate the size positively (Fig. 2). For the former, size was no issue, as the toilet was provided with a new superstructure. For the latter, size was difficult to evaluate on the basis of pictures alone. Therefore, the evaluation of this aspect should be based mainly on the comments of the one-time users, leading to the conclusion that the next version of the toilet should be smaller to fit into a larger number of existing superstructures.

### 4.3 Principle 3: convenient and aspirational design

We expected to collect a maximum of 2.1 kg of feces per day, and as the actual figure was up to 1.75 kg per day, it appears that most regular users preferred the toilet for defecating (H3a, Criterion 1) compared to using other sanitation facilities in the area. However, only up to 2.7 L of urine was collected (we expected a maximum of 10 L), which means that only a few regular users urinated in the BDT without defecating. At 40 L per day, the water consumption was also lower than the expected 75 L. However, considering the much lower frequency of use derived from the collected urine, water consumption per use was about twice as high as expected H3a, Criterion 2). This reconfirms the importance of the water-related features and that the recovered water was used in spite of the odor problem.

The usability features earned mostly positive evaluations, but with some critiques (Fig. 2). In particular, the foot pump received negative comments, though, still by < 20% of the participants (H3b). People expected that children as well as elderly and disabled persons would have difficulties using it. The same fear was expressed with respect to stepping up to the toilet and squatting.

### 4.4 Principle 4: establishing a financially sustainable business model

**Fig. 3:**
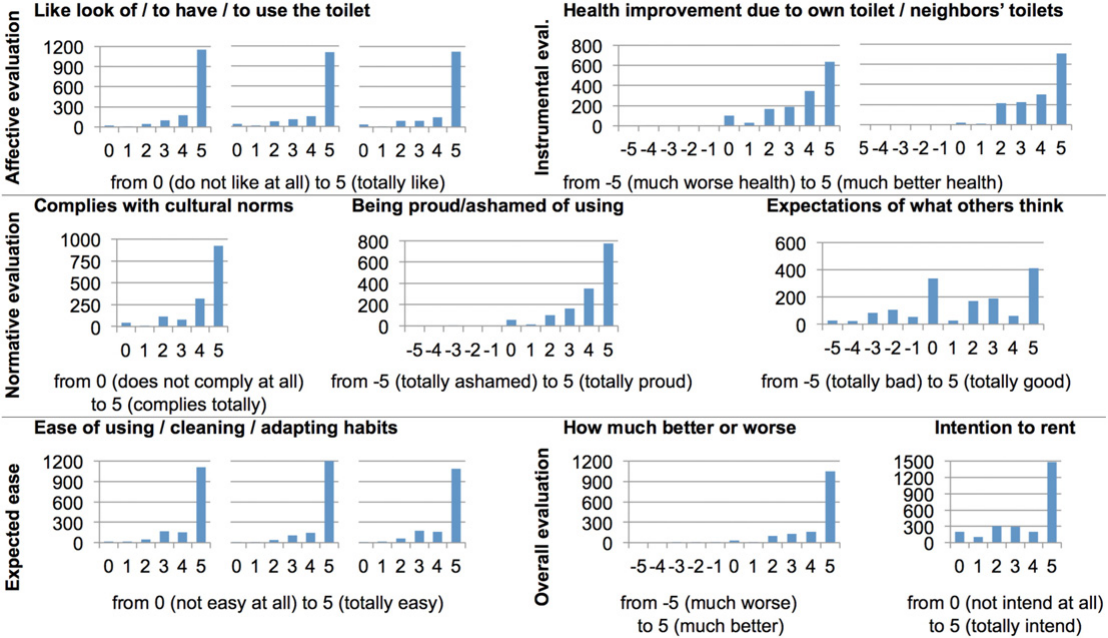
Frequencies of answers given to the closed questions by the survey sample. Note: Higher values represent a more favorable overall evaluation of the toilet.

The valuation of the BDT as assessed by the psychological determinant of the purchase decision is presented in Fig. 3 (see SI, Table S5, for the numerical values).

The evaluations were mostly positive (Fig. 3): the participants liked the toilet, social norms did not seem to present any obstacles, and participants expected to receive valuable health benefits and evaluated the toilet as being easy to integrate into daily life. Overall, the participants believed that their situation would be improved by having the toilet at home. The criterion of H4a was fulfilled by all evaluation dimensions, with one exception: the item regarding what others might think about the respondent if he or she had such a toilet. This item had only one-third of the responses in the highest two categories, and 42% of the responses were at zero or even negative values. This finding points to social processes that might be problematic for promoting the toilet and would require further investigation.

The positive overall evaluation was also reflected in the intention to rent the toilet, 65% of the answers being in the highest two categories. The criterion for H4b was also fulfilled: half of the respondents (51.4%) agreed to be placed on the bogus waiting list. As far as can be estimated this early in the process, therefore, there appears to be a market potential for the BDT.

## 5 Discussion

We tested nine hypotheses to investigate the feasibility and acceptance of four basic principles that we consider critical for an ecologically and economically sustainable toilet that the inhabitants of urban informal settlements are willing and able to use and pay for. By interviewing those who used the working model of the toilet for two weeks, a larger number of persons who used the working model once, and a representative survey that evaluated the toilet on the basis of information alone, we were able to create a picture that balances the validity and representativeness of the evaluations.

With regard to source separation and resource recovery (Principle 1), three aspects can be distinguished: (1) Separating urine and feces is feasible (H1a, Criterion 1, supported) and accepted in the target area (H1b supported except feces compartment); (2) separating water and urine turned out to be technically challenging due to mud in the water (H1a, Criterion 2, failed); (3) the feces compartment and collection service were not well received by the population (H1b failed for feces compartment). The general acceptance of urine source separation but with a critical attitude to its technical difficulties resembles earlier findings in Europe on urine-separating flush toilets (Lienert and Larsen, 2010). The problems of urine-water separation were responsible for three of five failed tests of the BDT (H1a, Criterion 2; H2a, Criterion 2; H2b, one-time users), but are not considered to be insurmountable. However, the size and proximity of the feces compartment to the user is a challenge for the technology. Although experience with UDDTs has shown that acceptance rises rapidly even after initial resistance when people realize their advantages (Uddin et al., 2014), traditional UDDTs have larger and more distant feces compartments, making it easier to ignore the fact that the feces are stored (Dellström Rosenquist, 2005). Since larger feces compartments are not possible in a dense urban slum environment, suitable technical solutions must be developed, e.g. improving the lid function with blinds. Fears that a collection service will be unreliable could also pose a serious barrier to purchase. Our own research (Schmitt et al., 2016) has shown that it is possible to plan and optimize a collection service. But more research in this neglected area is urgently needed.

The most controversial principle is the provision and on-site recovery of water for hand-washing and personal hygiene (Principle 2). Technically, the water recovery unit functioned without problems and produced hygienically flawless water (H2a, Criterion 1, supported). However, previous studies showed that the use of recycled water in showers and sinks is problematic, even though it is accepted for car washing and toilet flushing (Dolnicar and Schäfer, 2009). Our results indicate a pragmatic attitude toward water reuse: provided that the aesthetic quality of the water was good and the participants could be convinced of its hygienic quality, they accepted it (H2b supported by regular users and survey participants), and the provision of water proved a crucial selling point (H2c supported). The smell arising due to insufficient urine separation (H2a, Criterion 2, failed), however, was not well accepted and led to more negative evaluations (H2b for one-time users failed). Furthermore, the size of the water wall must be reduced. Generally, the study confirmed the viability of on-site water reuse from a toilet; to our knowledge, such a finding has not previously been documented in the literature.

The toilet was well used, particularly for defecating (H3a supported), and was evaluated as convenient and attractive (H3b supported). This indicates that the target population is open to new and unconventional designs. However, some room for improvement exists regarding its usability for children as well as elderly and disabled persons (e.g. replacing the foot pump by an electrical pump and lowering the platform). From a technical perspective, the primary use of the toilet for defecation may require a different distribution of storage volumes for feces and urine. It is likely that most people are not at home during the day and thus used the BDT only in the mornings, when defecation mainly takes place (Friedler et al., 1996), and evenings. It is also plausible that some outdoor urination takes place.

Finally, we estimated whether the toilet has a market potential (Principle 4). The psychological determinants are in favor of the decision to rent the toilet, with the least positive evaluation being what others might think of the participants if they rented and used it (H4a supported for all but the latter item). Although this indicates a small potential for normative problems such as jealousy or conflicts (Isunju et al., 2011), more than half of the participants decided on the spot to be put on the bogus waiting list (H4b supported). We expect that if more time were given to the participants to make the decision (so they could discuss it in advance with relevant people) and the toilet could be delivered within a short time (reducing the uncertainty regarding future constraints), more people would decide positively. However, this decision does not consider the constraints that the participants may ignore. Users who decide to rent the toilet may be unable to find space to set it up, may not get permission from their landlord to do so, or will set it up but then be unable to pay the rent (Scott et al., 2013). Nevertheless, we expect a substantial market potential for a toilet such as the BDT if the costs of the service can indeed be reduced to the level required by the Bill & Melinda Gates Foundation (i.e. US$0.05 per person per day). However, our analysis does not include the opportunity costs of not having the BDT (i.e. costs related to a higher likelihood of water-borne diseases).

On a more general level, this study shows how the basic principles of a research-driven innovation can be tested early in the development process and that the results can critically influence further developments. Field tests should thus be an integral part of the design process and not be used only to evaluate a final product. This philosophy is in line with the generally accepted participative approach to sanitation in low-income countries (Kennedy-Walker et al., 2014, Starkl et al., 2013). Combining qualitative and quantitative data from different samples with varying levels of interaction with the product leads to valid and representative data, even if only one working model is available for only a limited time.

Of course, the results of an investigation obtained so early in the development of a new technology should be interpreted with caution. During the development process, each new model must be re-evaluated so that the final product is still appropriate for potential customers. Potential uncertainties and biases also have to be considered. First, with any questionnaire-based data, there is the risk of a bias toward socially desirable answers (DeMaio, 1984). It is possible that less criticism and more praise were given to please the interviewers. However, the fact that some features were heavily criticized indicates that such a bias is, at least, not strong. Second, only a few people used the toilet regularly as part of their daily routine. This is the fundamental problem in testing early expensive working models. We addressed it by adding one-time users and a representative sample of people who evaluated the toilet on the basis of verbal and visual information. These evaluations have a lower validity than data from regular users, but, overall, this approach maximizes validity, reliability, and representativeness. Nevertheless, the results should be interpreted only as rough indicators, and field tests with a larger number of prototypes are needed in a later phase of development to generate statistically reliable data on consumer patterns and user satisfaction.

In conclusion, our approach allows us to generate a rough picture of how a product under development will be received by its users and what challenges have to be considered regarding its technical functioning, design, and promotion. Apart from being of fundamental importance for environmental protection and public health, the development of a new toilet comprises almost all the challenges a new technology could face at household level. We consequently believe that significant knowledge can be gained from this example about how innovative technologies can be tested at an early stage of their development, as well as how engineers and social scientists can cooperate to solve environmental problems.

## Figures and Tables

**Table 1:** Hypotheses tested and criteria used for testing them.

ID	Hypotheses	Criteria
H1a	Source separation is technically feasible in urban slums.	(1) No solid excreta in urine and water (2) No urine in water
H1b	Source separation and resource recovery are accepted.	< 20% of participants giving negative comments on source separation, pan and feces compartment, and emptying service
H2a	On-site water recovery for washing is technically feasible in urban slums.	(1) Unproblematic microbial quality and (2) No color and smell of the treated water
H2b	Recycled water for hand-washing and personal hygiene is accepted.	< 20% of participants giving negative comments on using recycled water
H2c	The provision of water for washing is an important selling point for new sanitation technologies in urban slums.	> 10% of participants giving positive comments on water-related features
H3a	The BDT is more attractive than any other sanitation option available to the participants and is therefore used for defecating and hand washing.	(1) Feces produced per day ~ 2 kg (2) Water used per session > 1.5 L
H3b	The design of the toilet is convenient for the users.	< 20% of participants giving negative comments on the usability of the toilet
H4a	Psychological determinants of purchase decisions are favorable.	(1) > 1/3 of answers in the highest two answering categories (2) < 1/3 of answers negative or neutral; for unipolar items, in lowest two categories
H4b	Simulated purchase decisions are positive for a sufficiently large percentage of people.	> 1/3 of participants decide to rent BDT

## Appendix A Supplementary data

Supplementary material Image 1
