# Supplementary material for: Early testing of new sanitation technology for urban slums: The case of the Blue Diversion Toilet
Source: Sci Total Environ. 2017 Jan 15;576:264–72. doi: 10.1016/j.scitotenv.2016.10.057 (PMC5179495; doi:10.1016/j.scitotenv.2016.10.057)
Supplement: Supplementary file 1 — Supplementary material [file mmc1.pdf]

SUPPORTING INFORMATION (SI)

Early testing of new sanitation technology for urban slums: the case of the Blue Diversion Toilet

Robert Tobias[a,1,*], Mark O'Keefe[a,2], Rahel Künzle[a], Heiko Gebauer[a], Harald Gründl[b], Eberhard Morgenroth[a,c], Wouter Pronk[a], Tove A. Larsen[a]

[1] Eawag, 8600 Dübendorf, Switzerland

[2] EOOS Design, 1010 Vienna/Austria

[3] ETH Zürich, 8093 Zürich, Switzerland

*Corresponding author
robert.tobias@uzh.ch

[1] Present address: University of Zurich, 8050 Zurich, Switzerland

[2] Present address: Sanergy, Nairobi, Kenya

1 Additional technical and economic information

1.1 Design and functionality of the Blue Diversion Toilet (BDT)

As stated in section 2 of the paper, the pan of the BDT allows for the separate collection of urine, feces and water. The liquid separation mechanism is based on electromagnetic valves controlled by electronics. By default, the urine valve is open and the water valve is closed, which changes if either the flush button is pushed or water flow at the shower head is detected. The water that is collected in the pump sump (compare Figure S1) is then recycled on site by a treatment system that consists of an ultrafiltration membrane bio reactor (Künzle et al., 2015) and a clean water tank for storing and polishing of the water. The goal of the polishing step is to reduce the color of and disinfect the water. The application of electrolysis is planned but could not be implemented in the present field study because more lab experiments are necessary to determine the optimal configuration regarding electrode material, size, voltage, electrode gap, and so on. Therefore, manual addition of chlorine to the clean water tank was implemented during the field study instead. For hygienic reasons, chlorine must be dosed or produced in sufficient amounts to secure a small residual chlorine concentration in the hand washing water. After use, this small residue is immediately consumed by the large amount of organic matter in the treatment reactor and thus poses no problems for the biological activity in this reactor.

The details regarding the water flow are presented in the two schemes in Figure S1. Comparing them shows that some pipes had to be implemented in the model to avoid an outflow of water to the environment in case one of the tanks overflowed. Sludge removal, which is shown in Figure S1 (b), is only required about yearly, if at all (Ravndal et al., 2015) and was never conducted during the field test. The recovery system was configured in modules in order to be able to add additional treatment steps if necessary, such as the granulated activated carbon filter between the treatment tank and the clean water tank, which was installed during the field study.

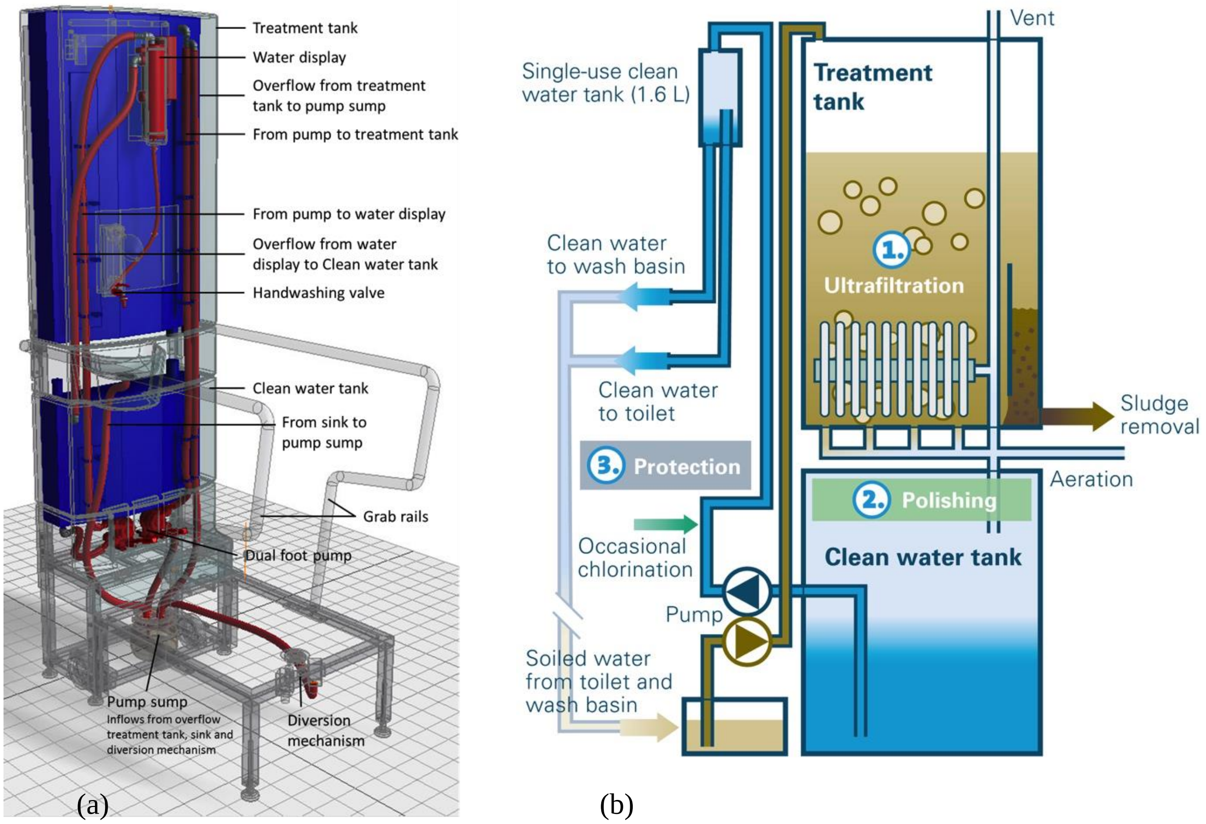

Figure S1: (a) Illustration of the hydraulic system, which is not visible to the users (all water pipes in red), and (b) the water recirculation system with the three barriers.

1.2 Maintenance during field test

The following maintenance activities were required during the field test:

- Optimizing the urine diversion mechanism
- Adding an activated carbon filter between the treatment tank and the clean water tank because of odor problems
- Repairing the feces cover
- Replacing the aeration pump

1.3 Measured parameters of water quality

To assess the water consumption, data loggers in the water tanks recorded the water pressure and temperature every five minutes. The water quality was assessed by means of a daily analysis of indicator bacteria (*E. coli*, total coliform, and total count) as well as free and total chlorine concentrations. Once a week, additional parameters of water quality were measured (see below). In addition, urine and feces were weighed when emptying the containers.

Table S1 compiles the parameters measured in the water from the shower head, the methods used for these measurements, and the average concentrations measured during the field test. These parameters were analyzed to monitor the treatment process and to ensure unproblematic water qual-

ity at all times. In addition, the water quality in the pump sump was also examined occasionally to judge how well the separation mechanism worked.

Table S1: Water quality parameters were measured daily and weekly in the water from the hand shower

Parameter	Method	Average concentrations
**Everyday**		
Indicator bacteria *E.coli*, total coliform and total count	Compact dry plates type EC and TC	*E.coli*: <1 CFU/100mL<br>Total coliform: 25 CFU/100mL<br>Total count: 4 CFU/100mL
Free chlorine and total chlorine	Hach Lange powder pillows	4.7 mg Cl_2/L and 8.2 mg Cl_2/L
Color	Hach Lange colorimeter	50 PtCo
**Once a week**		
Ammonia, nitrate, nitrite	Hach Lange powder pillow	135 mg NH_3-N/L,11.4 NO_3-N/L and 0.12 mg NO_2-N/L
Chemical oxygen demand (COD)	External lab	75.7 mg/L

CFU = colony forming unit; PtCO = Platinum-Cobalt scale

Of the measured parameters, only ammonia and nitrate were significantly higher than expected. Due to the problems with the separation unit mentioned in the paper, more urine ended up in the water recycling system, which elevated the ammonia concentration. A decrease in nitrogen can be seen when comparing the concentration in the pump sump with that from the shower head, but not all ammonia could be degraded. COD concentration, chlorine level, and color intensity were in the expected range, meaning the water was visually acceptable and residual chlorine was assured. This is confirmed by the low indicator bacteria counts. Although drinking-water quality is not necessary for handwashing, the achieved *E. coli* concentration mostly met the WHO drinking-water guidelines of 0 CFU/100mL and posed only a low risk (1-10 CFU/100mL) the rest of the time.

1.4 Business model calculations

The BDT is designed as a family-scale toilet but it is linked to a business model that includes the entire sanitation value chain, with a community-scale resource recovery plant (RRP) where resources from urine and feces are recovered. Thus, the business model considers not only the toilet itself but also the emptying and maintenance service and the resource recovery. The business model is based on the following assumptions:

- **Capital cost**: The capital cost consists of the production and distribution of the toilet and the RRP. The final version of the toilet is estimated to cost about US$500. Though, to reach this price, still large investments in the development of the toilet and a large market are required. The toilet can be used by 10 to 12 users for about 7 to 10 years. Thus, we assumed the capital cost of the toilet to be **US$0.014/user/day**.

The RRP serves 800 to 1,000 users for about 20 years and costs about US$27,500. Thus, for the RRP, we assumed the capital cost to be **US$0.004/user/day**.

- **Daily operating cost**: The operating cost consists mainly of the emptying service (logistics) and operating the RRP. For the toilet and the logistics, we assumed a cost of **US$0.024/user/day**, and for the RRP, we assumed a cost of **US$0.013/user/day**.
- **Business opportunity**: The business model assumes a **revenue of US$0.07/user/day**, which consists of the user fees of US$0.05/user/day and an assumed revenue from selling fertilizer produced in the RRP of US$0.02/user/day. Based on these assumptions, a business **profit of US$0.015/user/day** is possible.

The target market population was categorized into sanitation entrepreneurs and households. Sanitation entrepreneurs are private individuals who invest in a toilet and let neighbors pay to use it. These payments are then used to pay back the cost of the BDT. For the households, we assumed that an extended family or two families share the toilet. The toilet can be used about 50 times per day, and users can be family members or persons paying to use the BDT.

The product would be marketed through a franchising system. Franchisees sell the toilet to sanitation entrepreneurs and households and can be motivated to promote and sell toilets by providing a bonus system. Franchisees also maintain the toilet and collect the feces and urine. The franchising system was considered to have the lowest sales costs.

2 Additional information to methods

2.1 Measures taken to ensure the participants' comfort

The data gathering considered the sensitivity of the topic and was designed in close collaboration with local interviewers and a scientist from Makerere University. The wording of the questions focused on features and actions, not body parts or functions. All participants of a particular workshop were of the same gender, and the interviewers were always of the same gender as the interviewees. All interviews were done in private; the respondents' voluntary participation and right to refuse to answer the questions were emphasized. Of the persons contacted regarding the survey, 27% declined to participate, mostly due to a lack of time and apparently not because of the topic.

2.2 Information on the BDT provided to survey participants

The survey participants could not see or use the BDT before the interview. To allow them to evaluate the toilet, the interviewers explained the key features verbally and showed them pictures of the toilet. This information is compiled in the following sections.

2.2.1 General description

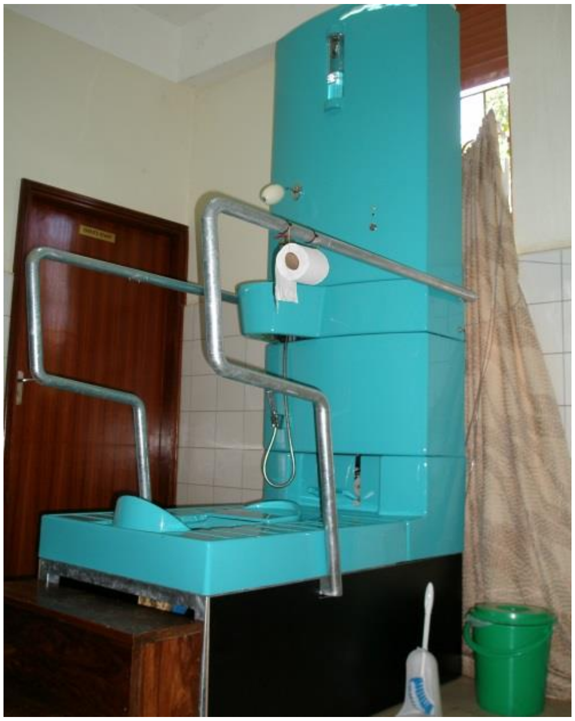

Figure S2. The complete Blue Diversion Toilet.

Down here is the toilet pan (Figure S2). You can use the toilet in the same way as you use your latrine. [Only for men: The toilet will also be equipped with a urinal for convenience.] Under the pan are containers that collect the urine and feces. To wash your bottom [only for women: or for menstrual hygiene], there is a hand shower, and the toilet also has a lavatory for washing your hands. Furthermore, after using the toilet, you can flush it so that the pan stays clean. The entire toilet is designed in a way that is very easy to keep clean. These tanks are perfectly closed and ventilated so that the toilet has no bad smells and no flies or vermin.

2.2.2 Urine diversion and resource recovery

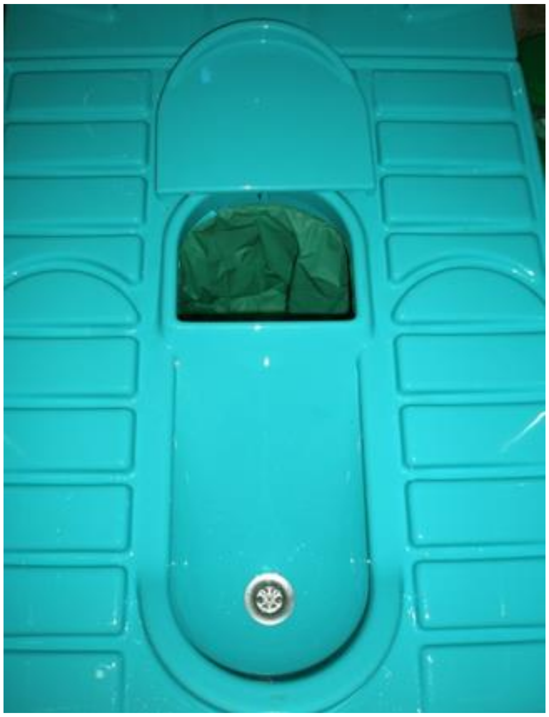

Figure S3. The Blue Diversion Toilet pan. Urine and feces are collected separately.

Here, you can see the pan a little better (Figure S3). You can see that feces and urine are collected separately. There is one container in the back for the feces, and the urine from the pan is collected in a separate container. This requires some small adaptations to your behavior. You cannot throw anything in the feces container and have to take care that not too much water or other liquids get in it. Thus, you have to open and close the container accordingly and get into the right position when using the toilet.

The urine and feces are collected in tanks that are emptied twice a week by a service included in the rent. The urine and feces will be transported to a local resource recovery plant, where fertilizer and distilled water are produced from the urine, using energy generated from the feces. The products will be sold to finance a portion of the toilet rent. However, the proceeds will not be enough to make the toilet free, and you will still have to pay rent for the toilet.

2.2.3 Water recovery

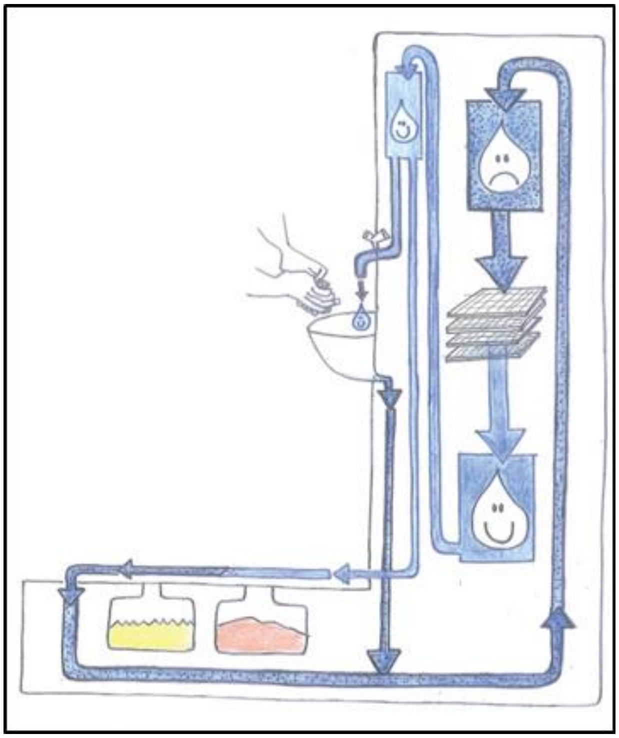

Figure S4. Schematic of the recovery of the water used for washing and flushing.

Now you might wonder where the water comes from. The water is recycled within the toilet (Figure S4). All the water you use for washing or flushing is cleaned with a powerful filter and is then perfectly safe to be used for washing, so you seldom have to refill the tank, and even then, only with very little water. The high-technology filter removes all dangerous substances and does not require any maintenance for many years (about 10). The filter is only a part of a more complex cleaning procedure, which additionally produces chlorine automatically. Thus, the water is chlorinated without the need to add any chlorine.

2.2.4 Further questions

If the participants had further questions, the interviewers answered them. However, at this point of the interview, no information on the price was given.

2.3 Details on the gathering of qualitative data

The first three principles investigated in the paper are about the acceptance and feasibility of the basic design principles that define the BDT. We considered open questions to be best suited to gathering qualitative data for the following reasons: First, when designing the study, we had no basis for determining what aspects of the toilet would be critical and on what dimensions the participants might evaluate these aspects. Therefore, it appeared best to let the participants evaluate the toilet in their own words and using their own logic. Second, any other procedure would lead to forced evaluations, meaning that participants who had not even noticed an aspect as special would have to give an evaluation of that aspect. This usually leads to a positive bias. Negative

opinions are the most noticed and therefore mentioned in the open-answer format. However, aspects that receive a positive evaluation when using forced-choice methods might have gone unnoticed by the participant and therefore might not enter into his/her actual evaluation of the toilet. For example, the soap holder was mentioned by very few participants, but mostly positively. We assume that when forced to evaluate the soap holder, most participants would have given a positive evaluation. Nevertheless, the fact that most participants did not mention it indicates that the soap holder in the current form is not an important selling point. Thus, with the approach used, the percentage of participants who mentioned certain features can be used as an indicator of the importance of the critique or praise. To avoid possible biases caused by the closed questions used in the interviews with the regular users and the survey participants, the qualitative evaluations were asked for before the quantitative evaluations.

There is, however, a drawback with this approach. It might happen that certain aspects are not mentioned because the participants think that they are not relevant for the study, or in the case of the long qualitative interviews done with the one-time users, they may have been forgotten. To reduce this bias (though increasing the risk of forced evaluations), the interviews of the one-time users were structured according to actions performed and some other aspects that might be relevant (see Section 2.3.2).

2.3.1 Regular users

The regular users were hand selected based on a number of criteria, mainly being close to the superstructure where the toilet was set up, being willing and able to use the toilet most of the time for two weeks, and being willing to be interviewed several times in this period. The gender and age composition of potential households was also typical for the target area. All participants were extensively informed about the project and the toilet, and the use of the latter was explained in more detail than to the one-time users, including aspects of maintenance (e.g., cleaning the toilet). All regular users were interviewed five times over the two weeks of using the toilet. After the instructions and the first use of the toilet, a short interview was conducted (about 15 minutes). During the usage period, three brief interviews were held (5 to 10 minutes each). After the last use of the toilet, a relatively long questionnaire was given (30 to 45 minutes). The questionnaires comprised mostly closed questions to investigate changes over time. However, these are not the topic of the paper presented, which focuses on comments on the toilet—namely, the features they liked and disliked and any particular difficulties they experienced. The question to assess this information was as follows:

> What do you like or dislike in particular about how the toilet looks and also about its features? Which feature do you like or dislike and why do you like or dislike it?
>
> What particular difficulties did you experience when using the toilet? How critical is this problem, and how could this problem be mitigated?

The participants could answer these questions completely freely and thus certain features were not mentioned at all.

2.3.2 One-time users

The one-time users were invited in groups of about 10 persons to participate in a workshop at the community center in Kifumbira. After a short introduction explaining the project, the toilet, and the workshop, each participant could use the toilet once in private. Afterwards, the participants were interviewed privately by an interviewer who noted their answers on a computer. Each interview took about 1 to 1.5 hours and consisted of four parts. Before and after the main part, a few closed questions were asked, mainly to assess some socio-demographic data and some items that were used in the questionnaire in the survey. These closed questions are not presented in the paper. After the private interviews, group discussions were held, mainly focusing on aspects of the business model and the service concept (e.g., the emptying service or the recovery of resources from urine and feces). The results of these discussions are also not presented in this paper. At the end, the participants could ask any questions they had and received compensation (half a kilo of sugar).

The main part of the semi-structured interviews of the one-time users was divided into two sections: First, the interviewers asked the participants to think about the entire process of using the toilet and report any experienced problems, particularly regarding the following actions: (1) stepping onto the toilet; (2) pumping the water; (3) opening the feces lid; (4) grabbing onto the hand rail; (5) positioning oneself over the pan/feces opening; (6) disposal of menstrual hygiene products; (7) cleaning the bottom; (8) standing up and turning around; (9) closing the feces lid; (10) flushing the toilet; (11) washing hands; and (12) stepping off the toilet. In the second part of the interview, the participants were asked to comment and evaluate each of the following features: (1) color of the toilet; (2) height of the toilet; (3) height of the step onto the toilet; (4) shape and size of the urine pan; (5) platform to stand on; (6) feces cover; (7) distance to feces; (8) volume of the feces container; (9) flush; (10) flush button; (11) hand rail; (12) soap holder; (13) hand wash basin; (14) tap for handwashing; (15) shower head; and (16) water recycling.

It is important to note that even though the interviewers used the mentioned actions and features to guide the interview, the participants were free in how they commented on their experiences. Certain actions and features might have received little attention and were not mentioned much, even if the interviewer reminded the participants about them, while other aspects were extensively commented on. However, the actions and features give the basic structure for the categorization of the comments by indicating what aspect a comment refers to. In a second step, it was determined whether the comment was positive, negative, or neutral, and then, what specific critique or praise was mentioned.

2.3.3 Survey participants

The survey participants were recruited by the random route procedure explained in the manuscript. If a person was willing and able to participate, a personal and private interview was performed at the home of the participant. The questionnaire comprised mostly closed questions. Those whose results are included in the manuscript are presented in Section 2.4. However, some open questions were also asked—most importantly, about what features they liked or disliked:

> What do you like or dislike in particular about how the toilet looks and also about the features I described to you? Which feature do you like or dislike, and why do you like or dislike it?

This question was asked directly after the toilet was presented through the verbal description and pictures (see Section 2.1). Again, the participants could freely answer, and it was possible that certain features were not evaluated at all (i.e., not mentioned as being liked or disliked).

2.4 Details on the gathering of quantitative data

Investigating the fourth principle cannot be done using qualitative data. Since the critical question to answer here is what behavior the participants might show in the future, it is necessary to gather data from constructs that are known to be related to behavior performance. We do not know what consequences a positive or negative evaluation of, for example, the water recovery has on the decision to rent the toilet. However, measures of attitudes, norms, and behavioral control have been proven to be correlated with the behaviors to which they relate. Since these constructs are predefined, a quantitative assessment of the answers is more efficient and informative.

The interviews with the survey participants lasted about one hour. The questionnaires comprised mostly closed questions, but also included some open questions about topics such as current sanitary situation; evaluations of the toilet regarding affective (liking), instrumental (health benefits), and normative considerations; expected problems and benefits; willingness to pay; alternative designs of the toilet and business model; and evaluations of specific features of the toilet (e.g., the water recovery). Table S2 compiles the wording and descriptive statistics of the closed questions whose results are presented in the paper. All items have been successfully used in a similar form in various other studies (Tobias and Berg, 2011; Tobias et al., 2013). Note that unfolding scales were used to gather the data. This means that the respondents were first asked to evaluate the question using only three response options (five in the case of bi-polar items). For example, after being asked questions regarding the affective evaluations, the participants had the answering options “don’t like,” “moderately like,” and “like very much.” After selecting one of these options, they were asked to specify their answer. For example, if a respondent answered “very like,” he or she was asked to specify whether this meant “mostly like” or “totally like.” This procedure leads to high resolution data without requiring the participants to memorize six (or, in the case of bipolar items, even 11) answering options. This way, it is possible to apply scales with high resolution even to participants with low formal education.

Table S2: Wording of the closed questions used in the survey and the descriptive statistics (mean and standard deviation) of these items.

Construct	Question	Response options	Mean	SD
**Affective evaluation**	How much do you like the way the toilet looks?	from 0 (do not like at all) to 5 (totally like)	4.70	0.73
**Affective evaluation**	How much do you like having such a toilet at you home?	from 0 (do not like at all) to 5 (totally like)	4.00	0.95
**Affective evaluation**	How much do you like using such a toilet regularly?	from 0 (do not like at all) to 5 (totally like)	4.00	0.94
**Normative evaluation**	Does the toilet comply with the cultur-al and religious norms of your house-hold?	from 0 (does not comply at all) to 5 (complies totally)	4.30	1.20
**Normative evaluation**	How proud or ashamed would you feel if you would use such a toilet at your home?	from -5 (totally ashamed) to 5 (totally proud)	4.10	1.30
**Normative evaluation**	What do you expect how good or bad other persons outside your household would think about you if you would have and use such a toilet at home?	from -5 (totally bad) to 5 (totally good)	1.70	2.80
**Instrumental evaluation**	How much do you think that the health of your household members decline or improve, if the diversion toilet would be available only in your household?	from -5 (much worse health) to 5 (much better health)	3.70	1.50
**Instrumental evaluation**	How much do you think that the health of your household members decline or improve, if the diversion toilet would be available in all households of your neighborhood?	from -5 (much worse health) to 5 (much better health)	3.90	1.30
**Expected difficulty[1)]**	What do you think, how difficult would it be for you and your family to use the toilet?	from 0 (not difficult at all) to 5 (totally difficult	0.51	1.00
**Expected difficulty[1)]**	Do you think cleaning this toilet would be difficult?	from 0 (not difficult at all) to 5 (totally difficult	0.37	0.85
**Expected difficulty[1)]**	How difficult do you expect it to be to adapt your habits from the latrine you use currently to using this toilet?	from 0 (not difficult at all) to 5 (totally difficult	0.54	1.01

**Overall evaluation**	What do you think, how much better or worse would it be for you having this toilet at home compared to the current state?	from -5 (much worse) to 5 (much better)	4.40	1.20
**Overall evaluation**	If the toilet would cost about 30,000 UGX per month including a service for emptying the containers twice a week, how much would you intend to rent the toilet?	from 0 (not intend at all) to 5 (totally intend)	2.80	1.70

1) The ease of using, cleaning, and adapting habits presented in the paper were actually assessed as expected difficulties. For Figure 3 of the paper, the labels and values were adapted to present the same data as ease instead of difficulties. Thus, the value 0 (not difficult at all) was changed to 5 (totally easy), the value 5 (totally difficult) to 0 (not easy at all), etc.

2.5 Further information to the bogus wait-list item

As explained in the paper, to investigate the market potential of the BDT, we did not want to rely only on measures of psychological evaluations and intentions. Even if the BDT was evaluated very positively, it is possible that nobody would rent it due to constraints related to the implementation of the intention. For example, people might be willing to pay the price but might not find the space to set up the toilet, or other persons that also have a say in the decision to rent the BDT might object. Of course, the only valid measure for market success is the number of people actually paying for the service. However, these numbers are only available after all the development efforts have been invested and the final product is available. In contrast, we want to determine a rough impression of the market potential long before the final version of the product is available on the market. By definition, such a forecast will never be as accurate as a post-hoc assessment of the actual market success. However, such estimates are available long before the main investments are done.

A common approach to estimating the market potential of a not yet existing product are measures of contingent valuation (Mitchell and Carson, 2005). The idea is to assess what value people assign to the future product and compare this to what it will actually cost. Directly asking the price one is willing to pay overstrains most participants, particularly those with low levels of formal education. Therefore, such data is considered as having low validity and reliability. Another approach is using ultimatum questions of the form "Are you willing to pay X for this product?" When X is varied between participants over a range of values, it becomes possible to estimate the average willingness to pay of the entire population based on the individual yes/no answers (see, e.g. da Piaxao et al. (2011), for a recent application in the sanitation sector).

Even though contingent valuation is a valuable approach for expressing the preference for a product in monetary terms, it is not suited for the purposes of this study. The valuation is investigated by psychological constructs (see Section 2.4 of this document). Even though they do not express *how much* people are willing to pay, they indicate *why* people are or are not willing to pay. The latter information is not only more informative for correcting fundamental design flaws, but there are also a number of fundamental problems related to the assessment of the willingness to pay for a new toilet: First, purchasing a toilet is not only a monetary issue. Even a person who is willing to pay a higher price than the toilet actually costs might not purchase the toilet because, for example, there is no space to set up the toilet or when it comes time to pay, he or she do not have the money available. Thus, at an early stage of development, it is not informative to know only the willingness to pay.

Second, the hypothetical nature of the question to assess the willingness to pay poses ethical and methodological problems. If the question has a factual appearance, the price might be the basis for comparisons. Participants faced with a low price might find the current prices for sanitation services (or the actual price of the toilet when it comes on the market) unjustified, while participants faced with high prices might find it unfair that they are asked to pay a higher price while, for example, their neighbor was offered a lower price. On the other hand, if the hypothetical nature of the question is made very clear, the risk is high that the data has low validity, in particular, that the willingness to pay is overestimated.

The goal of the item discussed here was not to assess the perceived value of the toilet but to estimate whether a person would actually rent the toilet or not. Even if a product is perceived as a bargain, people might not buy it due to the constraints mentioned above. Conversely, they might buy a product they would evaluate as overpriced just because there is no better option available. Of course, even if people rent the toilet and start paying for it, the business might fail because, for example, people do not pay the rent regularly. Thus, the decision to rent the toilet is also just a rough indicator of how much success the product might have in the target market. Nevertheless, the decision to rent considers at least some of the possible constraints. There is a higher chance that people will consider whether they would have the money to pay the rent, who they would have to ask permission to set up the toilet, and whether there would be space for a toilet. Still, many participants might not consider certain constraints and due to the high instability in informal urban settlements, they also might ignore some constraints. Thus, when estimating the market potential of a product based on purchase decisions, large uncertainties have to be considered. Nevertheless, such an estimate is more informative than one based on the mere evaluation of the product.

In the case of a non-existing product, the purchase decision cannot be assessed straightforwardly. The only way to assess the decision in this situation is by using deception: Participants have to be convinced that they actually must decide whether to trade money for a product or service. Since they knew that the product was not yet available, we used the concept of a wait list. Participants

had to decide whether they wanted to be put on the list, which meant that as soon as the toilet was available, they would receive it but also have to pay for it. Assuming that the participants believed in the existence of such a wait list, an actual purchase decision could be assessed. However, due to a number of factors, this measure can be biased:

- An interview that turns into selling a product might have been perceived as a scam. The participants might get the impression that the interview was all about selling them something and not about a scientific investigation. In this light, some participants might have refused whatever product is offered, leading to less people agreeing to be put on the wait list.
- The participants had to decide relatively quickly. Even though the interviewers allowed participants as much time to think about the decision as they wanted, still, the time was not enough to do, for example, more detailed calculations on the household budget or to discuss the issue with other persons within and outside the household. We assume that this led to more people declining to be put on the wait list.
- On the other hand, the lack of time and the already mentioned instable living conditions might have led participants to ignore critical constraints that might make setting up the toilet or paying the rent impossible. This might not have led so much to an overestimation of the actual purchase decision but to an overestimation of successful transactions that result in a functional toilet for which the rent is paid as agreed upon.

Overall, we expect that the item we used underestimates the percentage of people who actually would decide to rent the toilet but might overestimate the number of people who actually are able to set up the toilet and pay for it. Therefore, the data gathered with this item have to be interpreted as a very rough indicator with a high level of uncertainty, though it is still the best information available to estimate the market potential of a product in such an early stage of development.

Using deception is ethically problematic and whether such an approach is acceptable or not depends a lot on the culture of the participants. Therefore, we discussed this item with local experts and the interviewers and did extensive pretests that included discussing critical items with the participants. Further, the final version of the questionnaire also included a few questions for evaluating the interview items. We did not receive even one critical comment regarding the bogus wait-list item, even though some other items were heavily criticized (e.g., the question on age). Of course, we also received official ethical approval for the questionnaire, including the bogus wait-list item, from the University of Zurich.

The bogus wait-list item was placed close to the end of the questionnaire after a series of items asking about business aspects and intentions. Directly before the bogus wait-list item, we asked about the intention to rent the toilet. Since the answer to this question might be an anchor for answering the bogus wait-list item, we investigated the relation between these two items. It turned out that they are almost completely unrelated (a logistic regression analysis resulted in a

Nagelkerke pseudo R^2 of only 5.3%). The interviewers then explained that they would like to know whether the participant wanted to rent the toilet and posed the following question:

> Can I put you on a wait list for the toilet so that you will get the toilet and have to pay 30,000 UGX per month for it as soon as it becomes available?

The participant could only answer yes or no. After entering the answer, the interviewers made the following statement:

> Thank you for your answer. I have to admit that there is no wait list for the toilet because the toilet is still in development, and it will take years until it is ready to be distributed. With this question, we just wanted to see how committed you are.

The participants could then give any comments they had, which were reported by the interviewers, before continuing the interview. As mentioned before, at the end of the interview, the participants were explicitly asked to comment on the interview and any specific items if they felt uneasy with any of the questions or answers they gave (e.g., whether some items annoyed them or whether they had difficulties answering certain items).

The price of UGX 30,000 (US$11) stated in the question above is a rough estimate of what the actual cost for a household in the target area might be. The aim was to formulate the item in a way that it fit all households and remained as simple as possible. This guaranteed comparability and more reliable and valid data. However, from an economic perspective, the price might be different from what a household actually would have to pay.

The stated price was derived as follows: The business plan is calculated assuming a toilet as a unit (see Section 1.4 of this document). Thus, a household would have to pay US$0.05 per day for 10 users (i.e., US$0.5/day), even if it is much smaller. In the case of smaller households renting a toilet, the cost for having the toilet at home would be the same as for a household with 10 users. However, in an area where the percentage of users per toilet is much lower, the cost for the emptying service and the resource recovery would be distributed over more toilets. We assumed that in the target area, the average household size is five persons (in fact, the survey data revealed that on average 4.39 persons live in a household). Based on these assumptions, we did the following calculation:

Cost of toilet:	US$0.028/user/day	(every user has to pay for two users)
Cost of RRP:	US$0.004/user/day	(same as in the case of 10 users per toilet)
Daily operating cost:	US$0.037/user/day	(same as in the case of 10 users per toilet)
Total:	US$0.069/user/day	

➔ US$0.069/user/day * 5 users * 30 days = US$10.35/toilet/month = UGX 26,910

This value was then rounded up to UGX 30,000 (US$ 11.32) to get an easily communicable number and to avoid the appearance of high accuracy in the price estimate.

It is important to note that in contrast to the business-model calculation based on the assumption of 10 users/toilet (presented in Section 1.4 of this document), these calculations were done without investigating logistics concepts and possible consequences for the resource recovery. Thus, the price communicated in the survey has to be understood as a rough estimate.

2.6 Data analysis and sample quotes

All qualitative data presented in the paper were analyzed in the same form and using the same system of categories. The responses to semi-structured questions were coded using directed content analysis (Hsieh and Shannon, 2005). This method allows the coder to categorize the statements according to a predefined framework in which key concepts or variables are categories. We started the development of the category system by analyzing the responses of the one-time users. Here, the questions were directed and systematic in order for us to gain direct feedback about a particular action or feature from the user. In the first level, it was determined whether a comment was positive, negative, or neutral. Then, further categories indicated what aspects need to be improved on or what the participants specifically liked about a feature. Examples of positive and negative responses to the different features are compiled in Table S3.

Table S3: Examples of positive and negative comments given regarding different features.

Feature	Positive comments	Negative comments
**Urine pan**	It is good and prevents urine from spilling all over the place.<br>Its shape prevents urine from spilling.	It looks to be too small so it should be widened a bit.<br>The hole on the urine bowl for taking away the dirty water is too small.
**Feces container**	It is good. It is even hidden and no one can easily see it.<br>As feces do not mix with the urine, there is no smell.	Container seems to be small.<br>I feel it is small for the case of people with big families.
**Emptying service**	It cannot get full, since emptying is done twice a week.<br>It will prevent the toilet from smelling.	I hope you will not deceive us about emptying wastes.<br>Inconveniences a family because of emptying twice a week – I expect smell in my compound or house.
**Feces compartment lid**	It is good. It even makes the toilet more beautiful.<br>It is easy to use.	Feces cover did not close properly.<br>Hard for kids and elderly.
**Flush**	It cleans the urine and prevents bad smell.<br>I have really liked it.	The pressure for flushing is low.<br>The water is too little and it pours only on one side.

**Shower head**	It is very flexible and can turn to whatever angle one may like to take it.<br>It is easy to use.	The pressure of water is low, so it should be increased.<br>The hand shower pours out little water, so more pressure for it to pour enough water would be better.
**Hand wash basin**	It does not spill water all over the toilet.<br>It’s a reminder to wash hands.	It is so high that the young children may not reach it.<br>It’s a bit high. It should be lowered to favor the young.
**Soap holder**	Because it looks nice and attractive.<br>It will help people practice hand washing.	It is high for children.<br>Very high for short people to reach.
**Water recovery**	I like it because the water is cleaned and filtered.<br>It is quite unbelievable that recycled water is cleaned and used again, but still the whole process is interesting and good.	I do not like it in the sense that I wash my bottom from there and the water comes back.<br>It’s better to have a water tank than using recycled water.
**Size**	It is the first thing I liked on the toilet.<br>It uses small space.	It is long. It can’t fit in my house.<br>Our structures are short so it may not fit.
**Foot pump**	I thought it was hard but it was not hard when I tried to pump.<br>It was easy pumping.	The foot pump is a bit rigid and therefore a switch would be the best option.<br>It was not easy. You need to apply a bit of force.
**Stepping up the toilet**	Fine to me even when I am pregnant.<br>The steps are very strong. Even a big person can use them.	Did not like them at all. Put no steps.<br>For me an elderly, it’s a problem to step on them. If they can be made short, it will be good.
**Squatting / hand rails**	Squatting was not so hard because I grabbed the rails, so this helped me.<br>Squatting is good because where the feet are placed it is comfortable.	The old cannot easily squat, so a better way like a seat toilet should be made.<br>It is not easy especially for big people like me.

For quantitative data, only the value frequencies were calculated and plotted. Table S2 also presents descriptive statistics to all quantitative variables under the assumption of being metric measures.

3 Additional Results

3.1 Problems mentioned by the regular users

Regular users were also directly asked about problems they experienced when using the toilet. Because this information did not add to the information gathered by the open evaluations of the features, it was not presented in the paper. For completeness, it is presented here.

From 68 responses, 29 (43%) identified no difficulties, 13 (19%) were annoyed by the feces compartment lid falling off, 10 (15%) were unhappy with the water (particularly its odor), 5 (7%) found it difficult to use the foot pump, 5 (7%) had issues removing the mud that got stuck in the grooves, and 6 (9%) responses mentioned the height of the toilet as a potential problem.

3.2 Numerical values of Figures 2 and 3

Tables S4 and S5 compile the numerical values of the frequencies presented in the Figures 2 and 3, respectively.

Table S4: Percentages of positive (+) and negative (–) comments given about the toilet. The comments are grouped by hypothesis.

	Survey		1-time user		regular user	
	+	–	+	–	+	–
H1b: Comments on urine pan	13.7%	0.8%	30.0%	12.0%	2.7%	0.9%
H1b: Comments on feces container	9.9%	11.4%	28.0%	42.0%	2.7%	13.6%
H1b: Comments on feces compartment lid	17.3%	1.2%	14.0%	49.0%	5.5%	4.5%
H1b: Comments on emptying service	11.4%	12.9%				
H2b: Comments on water recovery	27.6%	7.2%	40.0%	34.0%	22.7%	13.6%
H2c: Comments on hand wash basin	19.1%	2.0%	38.0%	14.0%	16.4%	2.7%
H2c: Comments on soap holder	5.5%	0.8%				
H2c: Comments on shower head	13.1%	0.9%	50.0%	15.0%	10.9%	0.9%
H2c: Comments on flush	12.0%	0.1%	44.0%	8.0%	5.5%	1.8%
H2c: Comments on size	26.9%	6.4%	12.0%	33.0%	29.1%	6.4%
H3b: Comments on foot pump	3.6%	1.7%	41.0%	15.0%		
H3b: Comments on stepping up the toilet			27.0%	10.0%		
H3b: Comments on squatting / hand rails			32.0%	9.0%		

Table S5: Frequencies and percentages of answers given to the closed questions by the survey sample.

	-5	-4	-3	-2	-1	0	1	2	3	4	5	Sum
Affective evaluation: like how the toilet looks						21	5	49	102	176	1153	**1506**
[from 0 (do not like at all) to 5 (totally like)]						1%	0%	3%	7%	12%	77%	
Affective evaluation: would like to have the toilet						39	14	80	109	154	1110	**1506**
[from 0 (do not like at all) to 5 (totally like)]						3%	1%	5%	7%	10%	74%	
Affective evaluation: would like to use the toilet						38	10	94	95	148	1121	**1506**
[from 0 (do not like at all) to 5 (totally like)]						3%	1%	6%	6%	10%	74%	
Instrumental evaluation: health improvement due to own toilet	0	1	0	2	4	103	30	167	188	345	637	**1477**
[from -5 (much worse health) to 5 (much better health)]	0%	0%	0%	0%	0%	7%	2%	11%	13%	23%	43%	
Instrumental evaluation: health improvement due to neighbors' toilets	0	1	1	0	2	22	12	213	226	300	707	**1484**
[from -5 (much worse health) to 5 (much better health)]	0%	0%	0%	0%	0%	1%	1%	14%	15%	20%	48%	
Normative evaluation: complies with cultural norms						41	7	112	81	318	925	**1484**
[from 0 (does not comply at all) to 5 (complies totally)]						3%	0%	8%	5%	21%	62%	
Normative evaluation: ashamed / proud of using	0	0	3	0	0	59	15	100	166	354	778	**1475**
[from -5 (totally ashamed) to 5 (totally proud)]	0%	0%	0%	0%	0%	4%	1%	7%	11%	24%	53%	
Normative evaluation: expectations of what others think	27	22	83	105	55	335	27	169	189	60	413	**1485**
[from -5 (totally bad) to 5 (totally good)]	2%	1%	6%	7%	4%	23%	2%	11%	13%	4%	28%	
Expected ease to use						17	14	47	167	153	1110	**1508**
[from 0 (not easy at all) to 5 (totally easy)]						1%	1%	3%	11%	10%	74%	
Expected ease to clean						10	8	36	108	147	1199	**1508**
[from 0 (not easy at all) to 5 (totally easy)]						1%	1%	2%	7%	10%	80%	
Expected ease to adapt habits						12	18	65	173	160	1084	**1512**
[from 0 (not easy at all) to 5 (totally easy)]						1%	1%	4%	11%	11%	72%	
Overall evaluation: how much better or worse	0	0	1	4	2	34	10	97	133	158	1049	**1488**
[from -5 (much worse) to 5 (much better)]	0%	0%	0%	0%	0%	2%	1%	7%	9%	11%	70%	
Overall evaluation: intention to rent BDT						203	110	304	290	196	1477	**2580**
[from 0 (not intend at all) to 5 (totally intend)]						8%	4%	12%	11%	8%	57%	
Willing to be put on the (bogus) waiting list						722	764					**1486**
[0 = no; 1 = yes]						49%	51%					

4 Literature cited

da Piaxao, A.N., da C. Vieira, W., de Lima, J.E., 2011. Contingent valuation of improvement in basic sanitation services: a case study in Brazil. Economic and Environmental Studies 11(3), 213–234.

Hsieh, H.F., Shannon, S.E., 2005. Three approaches to qualitative content analysis. Qualitative Health Research 15(9), 1277-1288.

Künzle, R., Pronk, W., Morgenroth, E., Larsen, T.A., 2015. An energy-efficient membrane bioreactor for on-site treatment and recovery of wastewater. Journal of Water Sanitation and Hygiene for Development 5(3), 448-455.

Mitchell, R.C., Carson, R.T., 2005. Using surveys to value public goods. The contingent valuation method, Fourth printing. Resources for the Future, New York, USA.

Ravndal, K.T., Künzle, R., Derlon, N., Morgenroth, E., 2015. On-site treatment of used wash-water using biologically activated membrane bioreactors operated at different solids retention times. Journal of Water Sanitation and Hygiene for Development 5(4), 544-552.

Tobias, R., Berg, M., 2011. Sustainable use of arsenic-removing sand filters in Vietnam: Psychological and social factors. Environmental Science and Technology 45(8), 3260-3267.

Tobias, R., Huber, A.C., Tamas, A., 2013. A methodology for quantifying effects and psychological functioning of behavior-change techniques. SAGE Open 3(4), DOI: 10.1177/2158244013517245.
